# Longitudinal effects of childhood recreational gymnastics participation on bone development: The Young Recreational Gymnast Study

**DOI:** 10.3389/fendo.2025.1598344

**Published:** 2025-09-04

**Authors:** Marta C. Erlandson, Matthew S. Chapelski, Margo E. K. Adam, Alexandra J. Zaluski, Adam D. G. Baxter-Jones

**Affiliations:** ^1^ College of Kinesiology, University of Saskatchewan, Saskatoon, SK, Canada; ^2^ Faculty of Kinesiology, Sport, and Recreation, University of Alberta, Edmonton, AB, Canada

**Keywords:** recreational gymnastics, bone health, peripheral quantitative computed tomography, physical activity, childhood, adolescence

## Abstract

**Purpose:**

Previous research in the Young Recreational Gymnast Study (2006–2014) found bone benefits from involvement in recreational gymnastics during young childhood. The purpose of this study was to identify any longitudinal effects of recreational gymnastics exposure during childhood on adolescent bone health.

**Methods:**

For the present analysis, longitudinal data were available from 118 participants (66 female participants; 33 gymnasts) of the original 178 who were recruited and assessed on between one and five measurement occasions (median 3) between 2008 and 2020. Peripheral quantitative computed tomography (pQCT) scans were completed at both the distal and shaft sites of their non-dominant radius and tibia. Multilevel random-effects models were constructed to assess differences in the development of bone content, density, and estimated bone strength between those exposed and not exposed to recreational gymnastics while controlling for biological age, sex, body weight, limb length, and physical activity.

**Results:**

Individuals who were exposed to recreational gymnastics during childhood were found to have greater total area, total content, bone strength index, trabecular area, trabecular content, and trabecular density at the distal radius compared to physically active controls. There were no differences at the radial shaft, distal tibia, or tibial shaft.

**Conclusion:**

Involvement in childhood recreational gymnastics may provide long-term benefits at the distal radius as individuals enter young adulthood.

## 1 Introduction

Bone development during childhood is an essential part of growth and can impact a child’s risk of osteoporosis and life-threatening fractures as they age (1). Factors that influence bone development include the environment, genetics, and physical activity participation (2, 3). While the greatest predictor of bone health is genetics (3), physical activity is a modifiable factor that may have the greatest positive influence on bone mineral accrual (4, 5). For example, being physically active during childhood was found to result in an increase in boys’ and girls’ total body bone mineral content (BMC) by 9% and 17%, respectively, 1 year after peak growth in BMC, when compared to inactive peers (6). Additionally, childhood and adolescence have been shown to be the ideal time to optimize bone mineral accrual and increase peak bone mass.

Approximately 40% of an individual’s bone mass is acquired during a 5-year period in adolescence (7). More precisely, 22% of total body BMC is accrued 3 years surrounding a child’s peak height velocity (7). Furthermore, researchers have found a strong relationship between maturity and bone architecture, bone mineral density (BMD), and strength in both male and female adolescents (8). These findings suggest that physical activity may have the greatest impact on bone mineral accrual during the time around a child’s peak height velocity. While adolescence appears to be the premier time to accrue bone, studies have shown that childhood involvement in physical activity may have long-term bone health benefits. Gunter and colleagues completed a 7-month jumping intervention in children 9 years of age and found that 3 years after study completion, the intervention group had significantly higher BMC at the hip, lumbar spine, and total body when compared to controls (9). At follow-up 8 years later, it was found that the hip BMC remained significantly greater during adolescence (10). In alignment with Gunter and colleagues, we evaluated the influence of repeated impacts on children’s bone health using a recreational gymnastics model.

In terms of long-term bone health, participation in elite gymnastics has been shown to provide gymnasts with greater bone strength years after cessation of gymnastics participation (11, 12). However, elite gymnastics is not attainable for every child; thus, participation in recreational gymnastics has been studied and has also been found to improve bone health in young children (13–16). Elite gymnasts have been found to experience peak magnitudes of 3.6–10.4 times their body weight on their upper and lower limbs during standard training (17), while ground reaction forces from recreational gymnastics skills have been reported to vary from 1/3 to 6 times their body weight (18). Initially, in a study of recreational gymnasts [the Young Recreational Gymnast Study (YRGS)] between 4 and 6 years of age, we found that exposure to recreational gymnastics increased their total body BMC by 3% and their femoral neck BMC by 7% when compared to physically active controls using dual-energy X-ray absorptiometry (19). Additionally, in different cohorts, recreational gymnastics was shown to improve lumbar spine and forearm areal BMD (20) and distal radius BMC (16). Furthermore, using peripheral quantitative computed tomography (pQCT), the YRGS cohort had significantly greater total bone content (ToC), total bone density (ToD), total cross-sectional bone area (ToA), and estimated bone strength index (BSIc) during young childhood at the distal radius when compared to physically active controls (21, 22). Lastly, Dowthwaite et al. found that ex-gymnasts involved in premenarchal competitive gymnastics training for a minimum of 5 hours/week had radius bone size and strength that were at least 20% greater when compared to those of controls at both the distal and mid-shaft of the radius post-menarche (23). This suggests that involvement in gymnastics in the pre-pubertal years may have long-term bone health benefits.

Previous studies in the YRGS cohort have shown that childhood involvement in recreational gymnastics provides positive bone benefits at the distal radius in the short term. However, it is unknown if these benefits are still present in adolescence when bone accrual accelerates. Therefore, the purpose of the present study was to assess whether baseline bone health benefits observed in childhood recreational gymnasts are still retained after the children enter adolescence. We hypothesized that the difference observed in the recreational gymnasts would be maintained at the distal radius.

## 2 Methods

### 2.1 Participants

Participants were drawn from the YRGS conducted at the University of Saskatchewan between 2006 and 2020. The details of the YRGS have been previously published (19, 22). In brief, 178 participants (4 to 6 years of age) were recruited between 2006 and 2009 into a mixed longitudinal study examining the influence of early-life exposure to recreational gymnastics participation on body composition development. pQCT was acquired at the University of Saskatchewan and added to the study protocol in 2008. In 2008–2009, baseline pQCT measurements were obtained for 127 participants (68 female and 59 male participants), with 69 exposed to recreational gymnastics (35 female and 34 male participants) (71%) of the original sample (21). Thus, the data for this analysis span the 2008–2020 data collection periods. Recreational gymnasts had to participate in recreational gymnastics for at least 45 minutes/week for at least 4 months and were recruited from the recreational and precompetitive gymnastics programs at four gymnastics clubs in Saskatoon, Saskatchewan. The controls were recruited from other recreational sport programs at the University of Saskatchewan, such as swimming, soccer, and “sports ‘r’ fun” summer sport camps. Controls had no exposure to recreational gymnastics but did have exposure to other weight-bearing sports such as ice hockey, basketball, soccer, and volleyball. Thus, participants were divided into two groups: recreational gymnasts and controls. For the present analysis, participants were measured over a maximum of five visits between 2008 and 2020, for a total of 428 measurements. Participants were removed from the present analysis if they did not have full data at a measurement occasion, as well as if they had any condition that inhibited them from performing physical activity safely or any health condition that is known to affect bone development (i.e., heart disease or musculoskeletal disease). A total of 118 participants (66 female participants; 33 gymnasts) (93% of participants with a baseline pQCT scan) fulfilled these criteria and were measured between one and five occasions (median of three visits) for a total of 282 measurements. Informed consent was obtained from their parents or guardians, and verbal assent was obtained from children before study initiation. This study was approved by the University of Saskatchewan’s Biomedical Research Ethics Board.

### 2.2 Anthropometry

Anthropometric measurements included height, body weight and limb length. Demographics such as age and sex were also collected. Height and sitting height were measured using a wall-mounted stadiometer to the nearest 0.5 cm (Holtain Ltd., Crosswell, UK). Body weight was measured using a calibrated digital scale to the nearest 0.5 kg (Model 1631, Tanita Corp., Tokyo, Japan). Left tibia length was measured from the base of the medial malleolus to the superior margin of the medial epicondyle and left ulna length was measured from the distal tip of the styloid process to the proximal endplate using an anthropometric caliper (Rosscraft Lufkin, Canada). All measurements were taken twice, and if the difference was >0.4 (cm or kg), a third measurement was performed (24). Then, the mean or median was reported depending on whether two or three measurements were taken (24).

### 2.3 Biological age

A biological age, indicating years from peak height velocity (PHV), was estimated. Many equations can be used to predict when a child will or if they have attained their PHV. The most commonly cited equation is the Mirwald et al. equation (25). This tool is non-invasive and can be applied to both male and female individuals. The calculation only requires the participant’s sex, age, height, sitting height, and body weight to calculate years from PHV. A negative number indicates how many years away the child is from reaching their PHV. A positive number shows how many years past their PHV the child is. Finally, this equation is more accurate the closer the child is to attaining their PHV (25).

The equations are as follows:


$$\begin{array}{c} \text{Male Years from PHV}\\ =-9.236+\left(0.0002708^{*}{\text{Leg Length}}^{*}\text{Sitting Height}\right)\\ +\left(-0.001663^{*}{\text{Age}}^{*}\text{Leg Length}\right)\\ +\left(0.007216^{*}{\text{Age}}^{*}\text{Sitting Height}\right)\\ +\left(0.02292^{*}\text{Weight by Height Ratio}\right), \end{array}$$

$$\begin{array}{c} \text{Female Years from PHV}\\ =-9.376+\left(0.0001882^{*}{\text{Leg Length}}^{*}\text{Sitting Height}\right)\\ +\left(0.0022^{*}{\text{Age}}^{*}\text{Leg Length}\right)\\ +\left(0.005841^{*}{\text{Age}}^{*}\text{Sitting Height}\right)-\left(0.002658^{*}{\text{Age}}^{*}\text{Weight}\right)\\ +\left(0.07693^{*}\text{Weight by Height Ratio}\right). \end{array}$$

For each individual, an age from PHV was estimated at each measurement occasion, and the estimate closest to age at PHV was then used to represent a participant’s age at PHV across the study duration.

### 2.4 Peripheral quantitative computed tomography

pQCT was used to assess cross-sections of bone health for the non-dominant radius and tibia. If the participants had ever had a fracture in the non-dominant limb, then the dominant limb was scanned (XCT, Stratec Medizintechnik GmbH, Pforzheim, Germany). Two trained technicians performed scans with a voxel size of 0.4 mm and a scan speed of 20 mm/s for every site. For all sites, a scout view scan was first performed to visualize the distal growth plate and identify the medial point of the distal endplate, which is where the reference line was placed. Radius scans were conducted at the distal and shaft sites at 4% and 65% of the limb length, respectively, while tibia scans were conducted at the distal and shaft of the tibia at 4% and 66% of the limb length, respectively. Scans were analyzed using the Stratec software, Version 6.0, and standard variables at each site were reported according to manufacturer recommendations. At the distal sites (4% sites), bone was separated from the surrounding tissues using contour mode 1 with a threshold of 280 mg/cm^3^. This process allowed us to assess ToC (mg), ToD (mg/cm^3^), and ToA (mm^2^), as well as trabecular bone area (TrA; mm^2^), trabecular bone content (TrC; mg), and trabecular bone density (TrD; mg/cm^3^) at the distal radius and tibia. Additionally, BSIc (mg/mm^4^) was calculated (ToA × ToD^2^) to estimate the bone’s resistance to compression (26). At the shaft sites (65% radius and 66% tibia), separation mode 4 with an outer threshold of 280 mg/cm^3^ and an inner threshold of 540 mg/cm^3^ was used to separate bone and determine cortical bone content (CoC; mg/mm), cortical bone density (CoD; mg/cm^3^), cortical bone cross-sectional area (CoA; mm^2^), cortical thickness (CoTHK; mm), and polar stress strain index (SSIp; mm^3^), which is an estimate of bone’s resistance to torsion. Short-term precision (CV% root mean square) for repeated bone measurements has been reported previously from our group and ranged from 1.8% to 6.3% at the radius and tibia (27).

### 2.5 Physical activity

The Netherlands Physical Activity Questionnaire (NPAQ) asks parents to report their child’s current physical activity levels. It has questions about activity preferences and everyday activity choices rather than recalling physical activity levels. Questionnaire scores range from 7 (low physical activity levels) to 35 (high physical activity levels). The NPAQ has been documented to be a reliable and valid method to assess a child’s physical activity levels for children 4 to 7 years of age (28).

Additionally, the Physical Activity Questionnaire for Children (PAQ-C; 8 to 14 years of age) and for Adolescents (PAQ-A; 14 to 18 years of age) were used once participants were older. The PAQ-C and PAQ-A are nine- and eight-item questionnaires that ask children about how much physical activity they engaged in the previous 7 days (29). The only difference between the two questionnaires is that the PAQ-C asks about how active the child was at recess, while the PAQ-A does not ask about recess. Both give scores ranging from 1 to 5. A score of 1 means a child participated in a low amount of physical activity the previous week, while a score of 5 means a child participated in a high amount of physical activity the week prior. Both these tools have been found to be reliable and valid (30–32). We employed these different questionnaires due to the age-appropriate validation of the tools.

### 2.6 Statistical analysis

Descriptive statistics were analyzed by an independent t-test using SPSS (Version 29). For the longitudinal analyses, multilevel (hierarchical) random-effects models were constructed using a multilevel modeling approach (MlwiN Version 3.13, Centre for Multilevel Modelling, University of Bristol, UK) (33). A detailed description of the multilevel modeling procedures is presented elsewhere (34). In brief, bone parameters (ToA, ToC, ToD, TrA, TrC, TrD, BSIc, CoA, CoC, CoD, CoTHK, and SSIp) were measured repeatedly in individuals (level 1 of the hierarchy) and between individuals (level 2 of the hierarchy). Analysis models that contain variables measured at different levels of a hierarchy are known as multilevel regression models. Additive random-effects multilevel regression models were adopted to describe the developmental changes in bone parameters with biological age and are described in detail previously (22). Furthermore, since physical activity was measured using tools with different scales, a physical activity z-score was calculated and used in the model using the equation z-score = (x − µ)/σ, where x is the individual score, μ is the mean, and σ is the standard deviation.

Models were built in a stepwise procedure; i.e., predictor variables were added one at a time, and the log-likelihood ratio statistics were used to judge the fit of the model. Biological age was added as both a random variable (level 1) and a fixed variable. This permits individuals to have independent intercepts and slopes and a calculation of the intercept–slope covariance relationship. A significant biological age coefficient at level 1 of the models indicates that a bone measurement is increasing significantly at each measurement occasion within individuals. Significant coefficients at the individual variance matrix (level 2) in each model indicate that individuals have significantly different growth curves for bone measurements, in terms of both their intercepts and the slopes, and that there is a relationship between intercepts and slopes in the model. Fixed predictor variables were accepted as significant if the estimated mean coefficient was greater than twice the standard error of the estimate (SEE; i.e., *p*< 0.05). If the retention criteria were not met, the predictor variable was discarded. The power functions of biological age^2^ were introduced into the linear models to allow for the non-linearity of growth and were retained whether or not it was significant so as to shape the developmental curves. The predictor variable coefficients were used to predict bone development for ToA, ToC, ToD, TrA, TrC, TrD, BSIc, CoA, CoC, CoD, and SSIp with biological age, body weight, limb length, physical activity, sex, and group (gymnastics exposure versus no exposure) controlled in the prediction equations. A total of 11 independent multilevel (hierarchical) random-effects models were constructed for each bone parameter (ToA, ToC, ToD, TrA, TrC, TrD, BSIc, CoA, CoC, CoD, and SSIp).

## 3 Results

### 3.1 Participant characteristics

The anthropometric characteristics of children exposed and not exposed to recreational gymnastics from 5 to 19 years of age are displayed in **Table 1**. At 15 years of age, children not exposed to recreational gymnastics participated in more physical activity than children exposed to recreational gymnastics (*p* = 0.01). There were no other differences within any other age category (*p* > 0.05). The average recreational gymnastics participation was 45 minutes/week (median, 1.5 hours/week), while the years of exposure on average was 3 years (median, 2 years).

**Table 1:** Descriptive characteristics for children not exposed and exposed to recreational gymnastics.

Age	5	6	7	8	9	10	11	12
Not exposed								
N	10	12	23	33	28	13	19	4
Sex (male/%)	6 (60%)	6 (50%)	11 (48%)	15 (45%)	11 (39%)	4 (31%)	13 (68%)	0 (0%)
Height (cm)	111.89 ± 6.99	117.75 ± 5.62	124.26 ± 5.66	129.49 ± 5.88	133.11 ± 6.79	137.07 ± 8.58	144.42 ± 6.47	146.93 ± 10.35
Weight (kg)	20.83 ± 3.65	21.74 ± 2.55	26.71 ± 4.82	28.71 ± 6.54	30.09 ± 4.76	33.78 ± 9.18	38.17 ± 6.29	42.63 ± 7.93
BMI (kg/m^2^)	16.53 ± 1.08	15.67 ± 1.35	17.19 ± 2.04	16.95 ± 2.62	16.91 ± 1.71	17.75 ± 3.04	18.20 ± 1.97	19.61 ± 2.04
Bio Age (years)	−5.83 ± 0.71	−5.12 ± 0.66	−4.469 ± 0.88	−3.74 ± 0.74	−3.22 ± 0.76	−2.71 ± 1.06	−2.13 ± 0.92	0.23 ± 1.48
PA score	25.10 ± 2.81	23.75 ± 3.91	26.35 ± 2.93	25.30 ± 3.99	25.14 ± 3.96	26.85 ± 3.51	22.19 ± 8.967	21.03 ± 11.46
Exposed								
N	2	5	10	9	11	7	1	1
Sex (male/%)	0 (0%)	2 (40%)	3 (30%)	2 (22%)	3 (27%)	3 (43%)	0 (0%)	0 (0%)
Height (cm)	108.80 ± 6.79	118.18 ± 4.96	121.84 ± 6.96	127.43 ± 7.99	131.65 ± 7.12	139.62 ± 5.79	138.10	145.90
Weight (kg)	18.20 ± 2.26	23.64 ± 2.16	24.74 ± 4.79	26.16 ± 3.77	27.97 ± 3.31	33.67 ± 6.99	31.50	31.50
BMI (kg/m^2^)	15.35 ± 0.01	16.93 ± 1.28	16.53 ± 1.87	16.05 ± 1.17	16.10 ± 0.89	17.16 ± 2.79	16.52	14.78
Bio Age (years)	−5.45 ± 0.42	−5.02 ± 0.79	−4.35 ± 0.57	−3.66 ± 0.34	−3.25 ± 0.63	−2.94 ± 0.99	−1.84	−1.15
PA score	27.50 ± 0.71	24.80 ± 1.64	25.20 ± 2.09	26.11 ± 3.92	26.09 ± 2.95	25.71 ± 2.36	25.00	34.00
Age	13	14	15	16	17	18	19	
Not exposed								
N	9	20	15	10	10	6	8	
Sex (male/%)	3 (33%)	10 (50%)	6 (40%)	2 (20%)	3 (30%)	3 (50%)	5 (63%)	
Height (cm)	160.77 ± 10.68	163.96 ± 8.66	166.86 ± 8.35	167.30 ± 10.69	169.70 ± 8.74	173.08 ± 10.23	175.48 ± 7.01	
Weight (kg)	54.99 ± 16.0	53.09 ± 10.17	59.73 ± 11.71	61.60 ± 8.312	61.75 ± 8.089	69.62 ± 9.29	77.41 ± 14.70	
BMI (kg/m^2^)	20.94 ± 4.05	19.59 ± 2.55	21.32 ± 3.19	22.09 ± 3.24	21.47 ± 2.64	23.19 ± 1.77	25.01 ± 3.46	
Bio Age (years)	0.61 ± 1.23	0.80 ± 1.06	1.88 ± 0.99	2.89 ± 0.52	3.49 ± 0.67	3.72 ± 0.45	4.63 ± 1.04	
PA score	2.99 ± 0.33	2.76 ± 0.59	2.86* ± 0.62	2.57 ± 0.71	2.22 ± 0.87	2.79 ± 0.86	1.75 ± 0.73	
Exposed								
N	3	3	3	2	1	2	2	
Sex (male/%)	1 (33%)	2 (66%)	0 (0%)	2 (100%)	1 (100%)	0 (0%)	0 (0%)	
Height (cm)	166.13 ± 4.37	170.13 ± 11.76	162.40 ± 2.29	167.90 ± 15.27	187.85	168.63 ± 2.09	170.43 ± 3.36	
Weight (kg)	53.23 ± 7.51	57.90 ± 4.61	57.77 ± 5.42	58.90 ± 17.39	68.90	61.35 ± 1.77	57.20 ± 4.81	
BMI (kg/m^2^)	19.41 ± 3.81	20.07 ± 1.68	21.89 ± 1.85	20.59 ± 2.41	19.53	21.57 ± 0.09	19.74 ± 2.43	
Bio Age (years)	0.89 ± 0.867	1.17 ± 1.24	2.389 ± 0.13	1.44 ± 1.28	3.53	4.10 ± 0.23	5.189 ± 0.49	
PA score	2.87 ± 0.55	2.82 ± 0.32	1.74 ± 0.48	2.86 ± 1.29	1.00	1.87 ± 0.04	2.53 ± 0.70	

*indicates a difference between children not exposed to and exposed to recreational gymnastics within a given age category (bolded).

BMI, body mass index; Bio Age, biological age; PA, physical activity.

### 3.2 Distal radius (4% site)

**Table 2** displays the results from the multilevel model for the distal radius bone measurements of ToA, ToC, ToD, BSIc, TrA, TrC, and TrD. Specifically, exposure to recreational gymnastics was a significant contributor to greater ToA, ToC, BSIc, TrA, TrC, and TrD. No differences were found for ToD for children exposed to recreational gymnastics. Biological age^2^, sex, body weight, radius length, and physical activity contributed to the prediction of ToA, while biological age, biological age^2^, sex, body weight, radius length, and physical activity contributed to the prediction of ToC and TrC. Furthermore, the constant, biological age, biological age^2^, and body weight contributed to ToD. BSIc was contributed to by the constant, sex, and body weight, while TrA was predicted by biological age^2^, sex, body weight, radius length, and physical activity. Finally, the constant, biological age, and body weight predicted TrD.

**Table 2:** Multilevel regression models for total cross-sectional bone area (ToA), total bone content (ToC), total bone density (ToD), bone strength index (BSIc), trabecular area (TrA), trabecular content (TrC), and trabecular bone density (TrD) at the 4% distal radius site.

Distal radius (4%)							
Variable	ToA (mm^2^)	ToC (mg)	ToD (mg/cm^3^)	BSIc (mg/mm^4^)	TrA (mm^2^)	TrC (mg)	TrD (mg/cm^3^)
Fixed effects							
Constant	NS	NS	289.05 ± 28.11	14.82 ± 6.59	NS	NS	242.44 ± 33.11
Biological age	NS	−5.52 ± 0.79	0.94 ± 1.16	NS	NS	−2.52 ± 0.79	−6.71 ± 1.61
Biological age^2^	−0.34 ± 0.17	−0.23 ± 0.06	0.30 ± 0.11	NS	−0.64 ± 0.19	−0.23 ± 0.07	NS
Sex	−21.40 ± 6.67	−4.68 ± 2.49	NS	−2.57 ± 1.17	−16.51 ± 7.31	−4.68 ± 2.49	NS
Weight	2.14 ± 0.47	0.84 ± 0.17	1.14 ± 0.34	0.44 ± .81	1.91 ± 0.51	0.84 ± 0.17	1.18 ± 0.34
Radius length	0.73 ± 0.19	0.17 ± 0.07	NS	NS	0.81 ± 0.21	0.17 ± 0.07	NS
PA	7.13 ± 52.59	2.05 ± 0.96	NS	NS	7.80 ± 2.80	2.05 ± 0.96	NS
Gymnastic exposure	20.38 ± 7.39	9.44 ± 2.74	NS	4.51 ± 1.18	17.77 ± 7.81	9.44 ± 2.74	19.29 ± 6.52
Random effects							
Level 1							
Constant	1,022.14 ± 135.94	163.62 ± 21.11	285.55 ± 40.04	27.24 ± 3.59	1,239.69 ± 163.55	163.62 ± 21.11	762.94 ± 83.26
Level 2							
Constant	1,112.56 ± 251.88	111.61 ± 29.96	1,482.74 ± 233.84	45.18 ± 8.89	1,447.24 ± 320.52	111.61 ± 29.96	468.63 ± 111.43
Biological age	28.23 ± 9.42	2.63 ± 1.15	13.20 ± 3.84	0.81 ± 0.25	38.40 ± 11.96	2.63 ± 1.15	NS
Constant * Biological age	146.64 ± 40.49	14.95 ± 4.75	88.04 ± 24.63	5.62 ± 1.32	213.14 ± 53.18	14.95 ± 7.75	NS

All numerical values are reported as significant, *p* < 0.05 (mean > 2 * SEE). NS, not significant.

Fixed-effects values are estimated mean coefficients ± standard error estimate (SEE) of total cross-sectional bone area (ToA; mm^2^), total bone content (ToC; mg/mm), total bone density (ToD; mg/cm^3^), bone strength index (BSIc), trabecular bone area (TrA; mm^2^), trabecular bone content (TrC; mg/mm), and trabecular bone density (TrD; mg/cm^3^) at the distal radius (4% site). Random-effects values are estimated mean variance ± SEE. Biological age [at peak height velocity (PHV) = 0], radius length (cm), physical activity score (z-score), sex (male = 0, female = 1), and gymnastic exposure (0 = no exposure, 1 = exposed to gymnastics).

### 3.3 Radial shaft (64% site)

**Table 3** summarizes the multilevel model output for the radial shaft; exposure to recreational gymnastics was not significant for any bone measurement at the radial shaft. We found that biological age^2^, body weight, and radius length predicted ToA. The constant, biological age, and body weight were significant contributors to ToC and CoC. The constant, biological age, biological age^2^, body weight, and radius length were significant contributors to CoD, while the constant, body weight, and radius length predicted CoA. CoTHK was predicted by the constant, biological age, body weight, and radius length. Finally, SSIp was contributed to by biological age, biological age^2^, body weight, radius length, and physical activity.

**Table 3:** Multilevel regression models for cortical cross-sectional total bone area (ToA), total bone content (ToC), cortical bone content (CoC), cortical bone density (CoD), cortical bone area (CoA), cortical thickness (CoTHK), and polar stress strain index (SSIp) at the 65% radial shaft.

Radial shaft (65%)							
Variable	ToA (mm^2^)	ToC (mg)	CoC (mg/mm)	CoD (mg/cm^3^)	CoA (mm^2^)	CoTHK (mm)	SSIp (mm^3^)
Fixed effects							
Constant	NS	40.31 ± 6.97	43.00 ± 6.72	1,101.1 ± 53.29	30.52 ± 8.82	3.04 ± 0.28	NS
Biological age	NS	1.73 ± 0.39	1.89 ± 0.38	20.91 ± 2.68	NS	0.05 ± 0.02	6.29 ± 1.57
Biological age^2^	0.12 ± 0.05	NS	NS	−1.18 ± 0.22	NS	NS	0.37 ± 0.11
Sex	NS	NS	NS	NS	NS	NS	NS
Weight	0.31 ± 0.15	0.69 ± 0.08	0.70 ± .08	1.23 ± 0.53	0.52 ± 0.09	0.02 ± 0.001	1.58 ± 0.33
Radius length	0.47 ± 0.06	NS	NS	−0.95 ± 0.25	0.13 ± 0.04	−0.004 ± 0.001	0.67 ± 0.11
PA	NS	NS	NS	NS	NS	NS	2.89 ± 1.68
Gymnastic exposure	NS	NS	NS	NS	NS	NS	NS
Random effects							
Level 1							
Constant	85.77 ± 11.51	23.86 ± 3.21	21.66 ± 2.94	1,727.71 ± 227.96	44.45 ± 5.80	0.04 ± .001	287.97 ± 40.02
Level 2							
Constant	191.46 ± 33.65	64.49 ± 11.13	64.45 ± 11.03	808.55 ± 259.18	60.39 ± 12.04	0.06 ± 0.01	1,361.08 ± 220.99
Biological age	NS	0.56 ± 0.21	0.70 ± 0.22	32.64 ± 13.1	NS	0.02 ± 0.01	21.01 ± 4.98
Constant * Biological age	NS	4.86 ± 1.29	5.53 ± 1.35	NS	NS	0.01 ± 0.00	146.32 ± 29.68

All numerical values are reported as significant, *p* < 0.05 (mean > 2 * SEE). NS, not significant.

Fixed-effects values are estimated mean coefficients ± standard error estimate (SEE) of total cross-sectional bone area (ToA; mm^2^), total bone content (ToC; mg/mm), cortical bone content (CoC; mg/mm), cortical bone density (CoD; mg/cm^3^), cortical bone area (CoA; mm^2^), cortical thickness (CoTHK), and polar stress strain index (SSIp) at the radial shaft (65% site). Random-effects values are estimated mean variance ± SEE. Biological age [at peak height velocity (PHV) = 0], radius length (cm), physical activity score (z-score), sex (male = 0, female = 1), and gymnastic exposure (0 = no exposure, 1 = exposed to gymnastics).

### 3.4 Distal tibia (4% site)

**Table 4** shows the multilevel model for outputs for the distal tibia. Once again, exposure to recreational gymnastics was not significant for any bone measurement at the distal tibia. ToA was predicted by biological age^2^, sex, body weight, and tibia length, while ToC was predicted by the constant, biological age, biological age^2^, sex, body weight, tibia length, and physical activity. The constant, body weight, and tibia length were significant predictors of ToD. BSIc was contributed to by the constant, biological age, sex, body weight, tibia length, and physical activity. Sex, body weight, tibia length, and physical activity were predictors of ToA, while TrC was contributed to by biological age^2^, sex, body weight, and tibia length. Finally, TrD was predicted by the constant, body weight, and tibia length.

**Table 4:** Multilevel regression models for total cross-sectional bone area (ToA), total bone content (ToC), total bone density (ToD), bone strength index (BSIc), trabecular area (TrA), trabecular content (TrC), and trabecular bone density (TrD) at the 4% distal tibia site.

Distal tibia (4%)							
Variable	ToA (mm^2^)	ToC (mg)	ToD (mg/cm^3^)	BSIc (mg/mm^4^)	TrA (mm^2^)	TrC (mg)	TrD (mg/cm^3^)
Fixed effects							
Constant	NS	107.45 ± 30.53	355.05 ± 29.83	45.03 ± 139.66	NS	NS	310.09 ± 26.68
Biological age	NS	4.02 ± 1.60	NS	1.56 ± 7.51	NS	NS	NS
Biological age^2^	−1.71 ± 0.39	−0.49 ± 0.13	NS	NS	−1.74 ± 0.45	−0.55 ± 0.15	NS
Sex	−66.99 ± 16.91	−25.81 ± 6.73	NS	−7.75 ± 0.62	−51.29 ± 17.85	−16.18 ± 6.61	NS
Weight	4.79 ± 1.09	2.44 ± 0.36	1.46 ± 0.34	1.15 ± 1.71	3.90 ± 1.25	1.65 ± 0.42	0.75 ± 0.28
Tibia length	1.88 ± 0.25	0.21 ± 0.09	−0.35 ± 0.09	NS	2.13 ± 0.28	0.46 ± 0.10	−0.19 ± 0.08
PA	NS	4.97 ± 1.75	NS	2.14 ± 0.82	NS	NS	NS
Gymnastic exposure	NS	NS	NS	NS	NS	NS	NS
Random effects							
Level 1							
Constant	3,242.81 ± 440.75	295.98 ± 41.43	261.12 ± 36.87	66.6 ± 9.34	4,833.78 ± 641.44	573.49 ± 75.99	256.02 ± 34.93
Level 2							
Constant	14,865.6 ± 2,380.1	111.61 ± 29.96	1,493.01 ± 235.89	368.15 ± 58.13	17,299.64 ± 2,868.40	1,840.22 ± 306.24	721.08 ± 121.16
Biological age	174.82 ± 44.64	1,858.5 ± 286.7	13.06 ± 3.71	4.46 ± 1.10	219.32 ± 58.09	15.64 ± 5.26	NS
Constant * Biological age	1,503.26 ± 296.02	145.6 ± 31.43	89.98 ± 24.29	33.62 ± 7.05	1,895.37 ± 373.26	162.50 ± 35.53	NS

All numerical values are reported as significant, *p* < 0.05 (mean > 2 * SEE). NS, not significant.

Fixed-effects values are estimated mean coefficients ± standard error estimate (SEE) of total cross-sectional bone area (ToA; mm^2^), total bone content (ToC; mg/mm), total bone density (ToD; mg/cm^3^), bone strength index (BSIc), trabecular bone area (TrA; mm^2^), trabecular bone content (TrC; mg/mm), and trabecular bone density (TrD; mg/cm^3^) at the distal tibia (4% site). Random-effects values are estimated mean variance ± SEE. Biological age [at peak height velocity (PHV) = 0], tibia length (cm), physical activity score (z-score), sex (male = 0, female = 1), and gymnastic exposure (0 = no exposure, 1 = exposed to gymnastics).

### 3.5 Tibial shaft (65% site)

**Table 5** displays the outputs from the multilevel model for the tibial shaft. Like the radial shaft and distal tibia, exposure to recreational gymnastics was not a contributor to any bone measurements. ToA was predicted by the constant, biological age, biological age^2^, sex, body weight, and tibia length, while ToC was significantly contributed to by the constant, biological age, biological age^2^, body weight, tibia length, and physical activity. The constant, biological age, body weight, and tibia length contributed to CoC. CoD was predicted by the constant, biological age, biological age^2^, and tibia length, while CoA was contributed to by biological age^2^, body weight, and tibia length. Finally, the constant, biological age^2^, and body weight predicted CoTHK, while the constant, biological age, body weight, tibia length, and physical activity contributed to SSIp.

**Table 5:** Multilevel regression models for cortical cross-sectional total bone area (ToA), total bone content (ToC), cortical bone content (CoC), cortical bone density (CoD), cortical bone area (CoA), cortical thickness (CoTHK), and polar stress strain index (SSIp) at the 65% tibial shaft.

Tibial shaft (65%)							
Variable	ToA (mm^2^)	ToC (mg)	CoC (mg/mm)	CoD (mg/cm^3^)	CoA (mm^2^)	CoTHK (mm)	SSIp (mm^3^)
Fixed effects							
Constant	−163.88 ± 54.57	118.78 ± 32.09	48.71 ± 18.37	1,133.49 ± 43.29	NS	2.91 ± 0.36	−377.67 ± 165.57
Biological age	−18.91 ± 2.94	28.74 ± 2.12	5.29 ± 1.03	19.85 ± 1.84	NS	NS	43.48 ± 9.65
Biological age^2^	−2.43 ± 0.28	2.76 ± 0.19	NS	−0.67 ± 0.19	−0.22 ± 0.09	−0.01 ± 0.002	NS
Sex	−15.25 ± 9.46	NS	NS	NS	NS	NS	NS
Weight	3.69 ± 0.67	1.80 ± 0.43	3.39 ± 0.23	NS	2.99 ± 0.24	0.04 ± 0.004	22.54 ± 2.17
Tibia length	1.37 ± 0.17	0.29 ± 0.09	0.18 ± 0.05	−0.57 ± 0.13	0.36 ± 0.06	NS	2.98 ± 0.49
PA	NS	6.39 ± 2.25	NS	NS	NS	NS	21.41 ± 10.39
Gymnastic exposure	NS	NS	NS	NS	NS	NS	NS
Random effects							
Level 1							
Constant	1,792.71 ± 242.93	794.58 ± 106.58	134.21 ± 18.69	1,205.36 ± 150.95	155.13 ± 21.71	0.05 ± 0.01	11,967.43 ± 1,664.16
Level 2							
Constant	2,448.03 ± 516.54	2,988.88 ± 517.10	630.89 ± 102.58	599.82 ± 168.22	523.18 ± 90.26	0.19 ± 0.03	57,892.06 ± 9,451.28
Biological age	56.97 ± 18.19	144.07 ± 25.72	10.10 ± 2.36	NS	10.31 ± 2.53	0.002 ± 0.001	1,165.37 ± 249.94
Constant * Biological age	291.21 ± 80.22	656.61 ± 111.33	70.29 ± 14.01	−87.65 ± 20.47	59.93 ± 13.19	0.014 ± 0.004	7,579.32 ± 1,417.31

All numerical values are reported as significant, *p* < 0.05 (mean > 2 * SEE). NS, not significant.

Fixed-effects values are estimated mean coefficients ± standard error estimate (SEE) of total cross-sectional bone area (ToA; mm^2^), total bone content (ToC; mg/mm), cortical bone content (CoC; mg/mm), cortical bone density (CoD; mg/cm^3^), cortical bone area (CoA; mm^2^), cortical thickness (CoTHK), and polar stress strain index (SSIp) at the tibial shaft (65% site). Random-effects values are estimated mean variance ± SEE. Biological age [at peak height velocity (PHV) = 0], tibia length (cm), physical activity score (z-score), sex (male = 0, female = 1), and gymnastic exposure (0 = no exposure, 1 = exposed to gymnastics).

## 4 Discussion

The purpose of our study was to evaluate whether individuals who participated in recreational gymnastics during childhood had any bone health benefits in adolescence when measured by pQCT. We found that recreational gymnasts had significantly higher ToA, ToC, BSIc, TrA, TrC, and TrD at the distal radius but no difference at the radial shaft or tibia when compared to physically active controls. This is important, as it demonstrates that distal radius bone health can be improved in the long term by involvement in recreational gymnastics during young childhood, even when compared to children involved in other weight-bearing activities and sports. Previous research demonstrated that participation in elite gymnastics provides long-term bone benefits in female individuals (35–37); however, as previously mentioned, elite gymnastics participation is not attainable on a population level. To the authors’ knowledge, this is the first study to show long-term bone benefits from involvement in childhood recreational gymnastics in both male and female individuals.

The results from this study are aligned with those of previous research in the YRGS cohort, which found that children involved in recreational gymnastics during young childhood had significantly higher ToC, ToD, and BSIc in a baseline cross-sectional analysis (21) and higher ToA and ToC longitudinal during childhood (22) at the distal radius compared to controls; both studies also found no changes at the radial shaft or the tibia. Research from other cohorts of gymnasts has also found involvement in gymnastics to have positive effects on bone health. In contrast to our findings, other researchers have found differences at the shaft site in both the radius and tibia. A study analyzing bone health in non-elite female gymnasts aged 6 to 12 years found that they had higher ToA and SSI than controls but lower ToD at the ulnar shaft (38). Ward and colleagues (39) compared gymnasts and controls aged 5 to 14 years and found that gymnasts had greater CoA and SSIp at the 50% radius length as well as greater CoA, CoTHK, and SSIp at the 65% tibia site. Another study assessing pre-menopausal retired elite artistic gymnasts found that they had higher BMC, ToA, and BSIc at the distal radius and higher BMC, ToA, CoA, CoD, and SSIp at the radial shaft (40). Part of this could be due to muscle mass, as muscle area was significantly associated with improvements in cortical BMC, area, and SSIp (41). Another explanation may be that the control group in the current study was an active group involved in other sports, which loads the lower limb and potentially also the upper limb in some sports such as basketball and volleyball. Additionally, there may be a dose response to long-term bone benefits. Burt et al. found that female individuals engaging in more than 5 hours/week of non-elite gymnastics have greater bone health than female individuals engaged in under 5 hours/week of non-elite gymnastics (38). Additionally, another study found that years of training was positively correlated with cortical BMC, area, and thickness (41). To be included in the current study, individuals only had to be participating in recreational gymnastics for 45 minutes/week, and the median exposure throughout the study duration to gymnastics was 1.5 hours/week, which may not have been a high-enough dose of ground reaction force to elicit greater bone health benefits, as seen in other studies (38, 39).

The reason that the bone health differences may only have been maintained at the distal radius is that gymnastics is one of the few sports to put high-impact strains/loads on the forearm, while most other sports put strain/load on the lower body. In fact, gymnasts experience more ground reaction forces compared to other recreational athletes (42). The lack of a difference in any of the bone variables at the radial shaft is surprising given the aforementioned load that recreational gymnastics puts on the forearm; however, no differences in the shaft were observed in the earlier investigations of this cohort either (21, 22). This could be because the variables assessed at the distal radius and radial shaft were different, or the distal radius is more susceptible to changes from loading. Finally, since our controls were physically active and engaged in a similar amount of physical activity (as assessed by the NPAQ and PAQ-C/A), it can be assumed that our controls and recreational gymnasts are continuing to accrue bone with mechanical stimulus, which may also explain why no differences existed at the radial shaft and tibia bone sites. Additionally, increases in BMC at the distal site are thought to be due to axial compression, while those in the shaft sites are more responsive to bending forces from muscle activity (43). Therefore, recreational gymnastics skills may put more axial compression on distal sites and not enough bending force to elicit change at shaft sites. Finally, elite male gymnasts have peak load magnitudes of 4 and 10 times their body weight for their upper and lower bodies, respectively (17). However, it is unlikely that recreational gymnastics reaches the same peak loads, in turn reducing some of the impact that it may have on long-term bone development (17). Additionally, body weight may not be the only factor influencing impact forces, as other factors such as landing technique have been reported to influence ground reaction forces experienced (18). Despite this, the ground reaction forces experienced during recreational gymnastics participation were maintained into adolescence, highlighting the potential of this type of activity on long-term bone health at the wrist.

Our physical activity assessment shows that the current cohort was continuing their engagement in physical activity. When comparing our physical activity scores to those of the Saskatchewan Pediatric Bone Mineral Accrual Study, our participants would be average in their activity levels (44). Baxter-Jones and colleagues measured boys and girls at 1 year past their PHV and categorized their participants’ physical activity levels into three groups: inactive, average, and active (44). Our average PAQ-C score was 3, which would put our participants in the average physical activity level group. Therefore, the current participants would have continued to accrue BMC in the loaded bones and would have increased their BMC as seen in Bailey and associates’ study (6). In fact, physical activity predicts adolescents’ bone strength at 8% and 50% of their tibia length (45). Also, it has been found that physical activity engagement may be declining at approximately 5 to 7 years of age (46, 47), and participation in sports helps mediate this decrease (47). This may explain why there are no differences at the tibia since our control group was involved in sports and would load the tibia. However, recreational gymnastics, unlike most sports, loads the wrist. Therefore, we can assume that the difference at the distal radius is due to involvement in recreational gymnastics in young childhood and not from participation in other sports.

Finally, if involvement in recreational gymnastics only improves bone variables at the wrist, there is still clinical importance to increasing bone at that site. Childhood fracture incidence rates range from 20 to 36 per 1,000 (48, 49), with fractures at the radius and ulna making up 36% of upper body fractures (49). Recent research has shown that the highest fracture incidence rates continue to be at the distal forearm, clavicle, and distal humerus (50). Furthermore, with wrist fracture incidence rates slightly decreasing for women while remaining stable for men (51), it is important to strengthen the wrist during childhood to reduce the risk of fracture later in life. Additionally, the upper limb accounts for 65% of all childhood fractures and is a common site of fracture later in life (52–54). Therefore, participation in recreational gymnastics may provide a way to improve bone health during childhood and reduce the risk of wrist fractures later in life. Lastly, participating in recreational gymnastics has been shown to benefit bone health even in older adults (55–57), highlighting the potential of recreational gymnastics as a modality for bone health.

### 4.1 Strengths and limitations

A strength of this study is the use of pQCT to measure bone health instead of dual-energy X-ray absorptiometry (DXA). pQCT allows us to look at trabecular and cortical bone independently and together, which DXA is unable to do. Additionally, pQCT allows us to evaluate a three-dimensional perspective of the bone rather than a two-dimensional perspective. This was also the first study, to the authors’ knowledge, to examine the longer-term influence of recreational gymnastics participation on bone health.

A limitation of this study is, first, the variability when participants were tested. Not every individual was present at all five measurement occasions. Second, we were unable to conduct a sex-specific analysis due to our limited power. Additionally, the physical activity questionnaires did not specifically assess osteogenic loads of the activities that participants engaged in over the years. We were also unable to quantify the exact loads experienced during all the different recreational gymnastics classes, as they occurred at different locations and over 12 years. However, our group has previously quantified the loads experienced while performing different recreational gymnastics skills after observing the classes occurring at these different recruitment locations (18). Lastly, our control group was a physically active group rather than a sedentary control group. While this potentially limits our ability to assess the full influence of recreational gymnastics participation on bone health, we believe that the physically active control group allows us to highlight that, compared to an active group of peers, recreational gymnastics participation imparts a benefit above and beyond other forms of physical activity and sport participation at the wrist.

### 4.2 Future directions

Future research should be conducted comparing recreational gymnasts to sedentary controls, which would provide more insight into the potential benefits of involvement in recreational gymnastics during childhood. Furthermore, studies should examine if there is a dose-response threshold for recreational gymnastics benefits on bone health.

### 4.3 Conclusion

In conclusion, individuals who were involved in recreational gymnastics during childhood had higher ToA, ToC, BSIc, TrA, TrC, and TrD at the distal radius but no difference at the radial shaft or the tibia when compared to physically active controls. Should this difference progress into adulthood, it may reduce the risk of obtaining a wrist fracture. Previous research in the same cohort has shown that there are short-term benefits from being involved in recreational gymnastics during childhood; however, this analysis adds to the knowledge base by extending the results through adolescence up to 19 years of age. This study highlights the long-term impact of early childhood exposure to weight-bearing activity, such as recreational gymnastics participation.

## Data Availability

The raw data supporting the conclusions of this article will be made available by the authors, without undue reservation.
